# Differences in outcomes between oral anticoagulation “new starters” and “switchers” in patients with nonvalvular atrial fibrillation: A pooled analysis of the AMADEUS and BOREALIS trials

**DOI:** 10.1002/joa3.12255

**Published:** 2019-11-11

**Authors:** Ying Bai, Alena Shantsila, Gregory Y. H. Lip

**Affiliations:** ^1^ Liverpool Centre for Cardiovascular Science University of Liverpool and Liverpool Heart & Chest Hospital Liverpool United Kingdom; ^2^ Cardiovascular Center Beijing Tongren Hospital Capital Medical University Beijing China; ^3^ Aalborg Thrombosis Research Unit Department of Clinical Medicine Aalborg University Aalborg Denmark

**Keywords:** new starter, switcher, vitamin K antagonists

## Abstract

**Background:**

To explore differences in outcomes between dose‐adjusted vitamin K antagonists (VKAs) “new starters” and “switchers” in patients with nonvalvular atrial fibrillation (AF).

**Methods:**

A post hoc analysis was performed to assess the outcome differences between VKA “new starters” and “switchers” in AF patients using pooled individual patient data of AMADEUS and BOREALIS trials.

**Results:**

A total of 4169 AF patients were included in the present analysis, which included 1383 “VKA new starters” and 2786 “VKA switchers”. VKA new starters had higher crude rates of all‐cause mortality (*P* = .035) and cardiovascular death (*P* = .047) compared to switchers. On multivariable Cox regression analysis, both “new starters” and “switchers” showed nonsignificant trends for different risks of stroke/systemic thromboembolism (SE) (hazard ratio (HR): 1.66, 95%CI: 0.95‐2.90, *P* = .08), major bleeding (HR: 1.25, 95% CI: 0.73‐2.16, *P* = .42), and all‐cause death (HR: 1.09, 95% CI: 0.75‐1.57, *P* = .65). On Kaplan‐Meier analysis, both groups had similar risks of stroke/systemic embolism (*P* = .09), major bleeding (*P* = .28), and all‐cause death (*P* = .06).

**Conclusions:**

In this post hoc analysis of clinical trial patients with AF, “new starters” and “switchers” for VKA initiation had nonstatistically significant rates of trial‐adjudicated thromboembolism, major bleeding, and all‐cause mortality.

## Graphical abstract

A post hoc analysis was performed to assess the outcome differences between VKA “new starters” and “switchers” in AF patients using pooled individual patient data of AMADEUS and BOREALIS trials. On Kaplan‐Meier analysis, both groups had similar risks of stroke/systemic embolism (*P* = .09), major bleeding (*P* = .28), and all‐cause death (*P* = .06). In this post hoc analysis of clinical trial patients with AF, “new starters” and “switchers” for VKA initiation had nonstatistically significant rates of trial‐adjudicated thromboembolism, major bleeding, and all‐cause mortality.

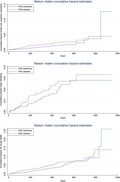

## 1 INTRODUCTION

Despite the non‐vitamin K antagonist oral anticoagulants (NOACs) being introduced for stroke prevention in atrial fibrillation (AF) in recent years, the vitamin K antagonists (VKAs) are still used as oral anticoagulants (OACs) in large number of patients.^1^, ^2^ Switching from one OAC drug to another is common, especially for VKAs to be changed to NOACs.^3^, ^4^, ^5^, ^6^ However, there is lack of evidence from direct comparisons on the effectiveness and safety of VKAs in those who have VKAs initiated following previous use of other OACs (ie, “switchers”) and in those who are newly started on VKAs (“new starters”).

The effectiveness and safety of different modes of VKA initiation (ie, VKA “switchers” vs VKA “new starters”) have been based on evidence from “real‐world” studies^7^, ^8^ and meta‐analyses.^9^ Patient willingness and compliance as well as physician adherence and compliance may impact on outcomes.^10^, ^11^

The aim of this analysis was to explore the differences in outcomes between VKA “new starters” and “switchers” in patients with nonvalvular AF. We performed a post hoc ancillary analysis using pooled individual patient data from two randomized, open‐label trials (AMADEUS and BOREALIS), with negligible subjective intention of switching between OACs and trial‐adjudicated outcomes.

## 2 METHODS

The full details of the designs of the AMADEUS and BOREALIS trials have been described previously, and are summarized in Supplemental Methods.^12^, ^13^ The AMADEUS and BOREALIS trials were approved by the institutional review boards and all patients provided written informed consent. Patients from the VKA arms were categorized into two groups according to their treatment after randomization, that is, “*VKA new starters”* (on VKA and without previous VKA treatment) or “*VKA switchers”* (on VKA and with previous VKA treatment).

### 2.1 Study endpoints

In this pooled analysis, we included all outcomes collected from the initiation of the treatment to the end of the studies. The primary analysis of the both trials reported only outcomes collected during the on‐treatment period. The primary efficacy outcome of this analysis was the composite of stroke and systemic thromboembolism (SE). Stroke was further classified into ischemic and nonischemic stroke based on brain imaging results. SE was confirmed by angiography, surgery, or autopsy in the original trials. The primary safety outcome was major bleeding, as defined on previously published criteria.^12^, ^13^ Other efficacy outcomes of venous thromboembolism, myocardial infarction, and safety outcomes of any clinically relevant bleeding, intracranial hemorrhage, were defined according to the original trials.^12^, ^13^ All‐cause death and the subgroups of fatal stroke and cardiovascular death were also assessed in this analysis.^12^, ^13^

Only the first event of each outcome and their occurrence date were used for analysis. Suspected outcome events in both trials were adjudicated by each independent central adjudication committee that was blinded to the treatment assignment.

### 2.2 Statistical analysis

Baseline characteristics were reported as percentages or mean ± SD. Data unavailable when merging the two datasets were treated as missing data. Comparisons of the continuous variables were performed using independent *t* test. Categorical variables were compared using Chi‐squared test or Fisher exact test.

Outcomes were expressed by event rate (number per 100 patient‐years). Two Cox proportional hazard models were used to estimate the hazard ratio (HR) for the outcomes of stroke/SE major bleeding and all‐cause death according to stratification of VKA switcher/new starter. Model I was adjusted for sex, age, diabetes mellitus, congestive heart failure and/or left ventricular impairment, previous stroke, hypertension, renal function (baseline creatinine clearance), suboptimal time in therapeutic range (TTR < 60), and concomitant aspirin use, to account for the main parameters known to effect the efficacy and safety of oral anticoagulation. Model II was adjusted for the demographic parameters, which were significantly different between groups, sex, AF type, body mass index, hypertension, congestive heart failure and/or left ventricular impairment, symptomatic coronary artery disease, renal function (baseline creatinine clearance), TTR, concomitant aspirin use, and concomitant clopidogrel or ticagrelor use. Nelson‐Aalen estimates were used for the two groups comparisons and then Kaplan‐Meier curves were assessed using a Log‐rank test for the two‐group comparison. A two‐tailed *P* value of <.05 was considered statistically significant. Analyses were performed using SPSS (version 23) and Stata (Version 13.0).

## 3 RESULTS

After merging the AMADEUS and BOREALIS datasets, 4169 AF patients treated with VKA were available for analysis, including 1383 “VKA new starters” and 2786 “VKA switchers”, with the follow‐up of 365 ± 187 days. In the AMADEUS dataset, mean follow‐up was 340 ± 165 (median 365[189‐460]) days and 396 ± 207 (median 399[211‐539]) days in BOREALIS dataset.

In the “VKA new starters” group, there were significantly more females, and paroxysmal AF, prevalent hypertension, left ventricular dysfunction, coronary artery disease concomitant aspirin, clopidogrel, or ticagrelor use. “VKA new starters” had significantly lower body mass index, creatinine clearance, and TTR (Table 1). The characteristics of the patients by AMADEUS and BOREALIS datasets were not different to the pooled analysis (Table SI).

**Table 1:** Baseline characteristics of VKA users

	Starter	Switcher	*P* value
Total patients	1383	2786	NA
Age (years)	69.59 ± 9.84	69.71 ± 9.17	.69
Categorized by group			.12
Age ≥75	473 (34.20)	922 (33.11)	
Age 65‐74	497 (35.94)	1090 (39.14)	
Age <65	413 (29.86)	773 (27.76)	
Gender female	592 (42.8)	938 (33.7)	<.001
AF type			<.001
Paroxysmal AF	479 (35.0)	830 (29.8)	
Persistent AF	192 (14.0)	356 (12.8)	
Permanent AF	699 (51.0)	1598 (57.4)	
BMI (kg/m^2^)	28.95 ± 5.61	29.55 ± 5.98	.002
Hypertension	1233 (89.2)	2295 (82.4)	<.001
Prior TIA/Stroke/SE	386 (27.9)	740 (26.6)	.36
Diabetes mellitus	335(24.2)	745 (26.7)	.08
LV dysfunction	680 (49.2)	959 (34.4)	<.001
Coronary artery disease	552 (42.2)	933 (34.1)	<.001
Concomitant treatment			
Aspirin	598 (43.2)	390 (14.0)	<.001
Clopidogrel or Ticagrelor	45 (3.3)	30 (1.1)	<.001
Other antiplatelet	10 (0.7)	10 (0.4)	.17
TTR (%)	51.86 ± 22.49	60.08 ± 21.46	<.001
Baseline creatinine clearance(ml/min)	87.78 ± 40.85	91.36 ± 29.92	.001
Categorized by group			
<30	0 (0)	5 (0.18)	<.001
30‐50	146 (10.66)	163 (5.90)	
50‐80	471 (34.40)	819 (29.66)	
≥80	752 (54.93)	1774 (64.25)	
CHA_2_DS_2_‐VASc score	4.07 ± 1.51	3.70 ± 1.52	<.001
0	0 (0)	0 (0)	<.001
1	43 (3.1)	158 (5.7)	
2	167 (12.1)	476 (17.2)	
3	297 (21.6)	691 (24.9)	
4	358 (26.0)	660 (23.8)	
5	270 (19.6)	458 (16.5)	
≥6	240 (17.5)	331 (12.0)	
HAS‐BLED score	2.14 ± 1.04	1.75 ± 0.98	<.001
0	61 (4.4)	241 (8.7)	<.001
1	311 (22.7)	922 (33.2)	
2	529 (38.5)	1029 (37.1)	
3	337 (24.5)	474 (17.1)	
4	119 (8.7)	98 (3.5)	
≥5	16 (1.2)	9 (0.3)	

Note

Data were presented as mean ± SD or percentage of actual patients number.

Abbreviations: AF, atrial fibrillation; BMI, body mass index; CHA2DS2‐VASc, congestive heart failure, 1 point; hypertension, 1 point; age ≥75 years, 2 points; diabetes mellitus, 1 point; stroke, 2 points; vascular disease, 1 point; age from 65 to 74 years, 1 point; and female sex, 1 point; HAS‐BLED, uncontrolled hypertension (>160 mmHg systolic), 1 point; abnormal renal function, 1 point; or abnormal liver function, 1 point; Prior history of stroke, 1 point; Prior major bleeding or predisposition to bleeding, 1 point; labile international normalised ratio, 1 point; age >65 years, 1 point; prior alcohol or drug usage history (≥8 drinks/week), 1 point; medication usage predisposing to bleeding: (Antiplatelet agents, NSAIDs), 1 point; LV, left ventricle; SE, systemic thromboembolism; TIA, transient ischemic attack; TTR, time in therapeutic range; VKA, vitamin K antagonists.

### 3.1 Efficacy and safety outcomes

“VKA new starters” had higher crude rates of all‐cause mortality (*P* = .036) and cardiovascular death rates (*P* = .047) compared to “VKA switchers” (Table SII).

**Figure 1:**
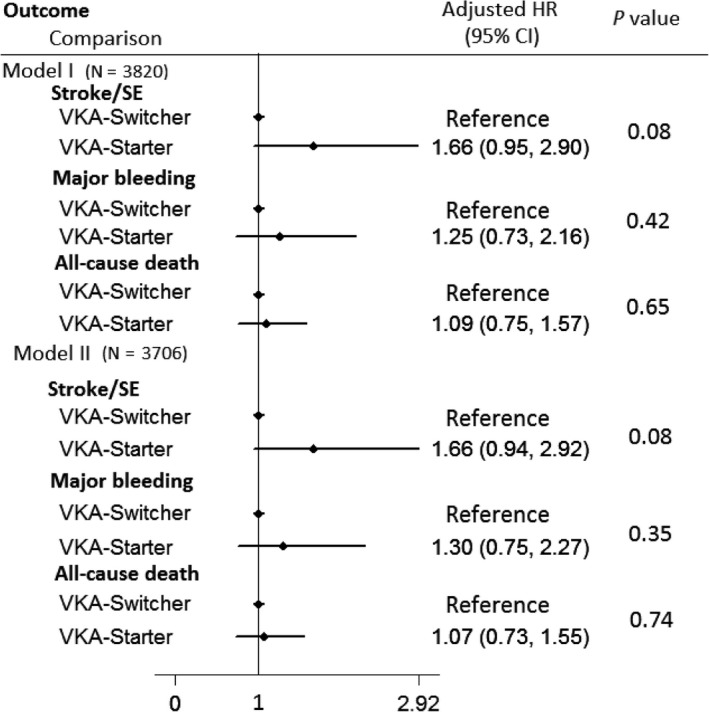
Forest plots for switchers and new starters comparison in the outcomes of stroke/SE, major bleeding, and all‐cause death. SE, systemic thromboembolism; VKA, vitamin K antagonists. See Statistics for covariates used in Model I and Model II

**Figure 2:**
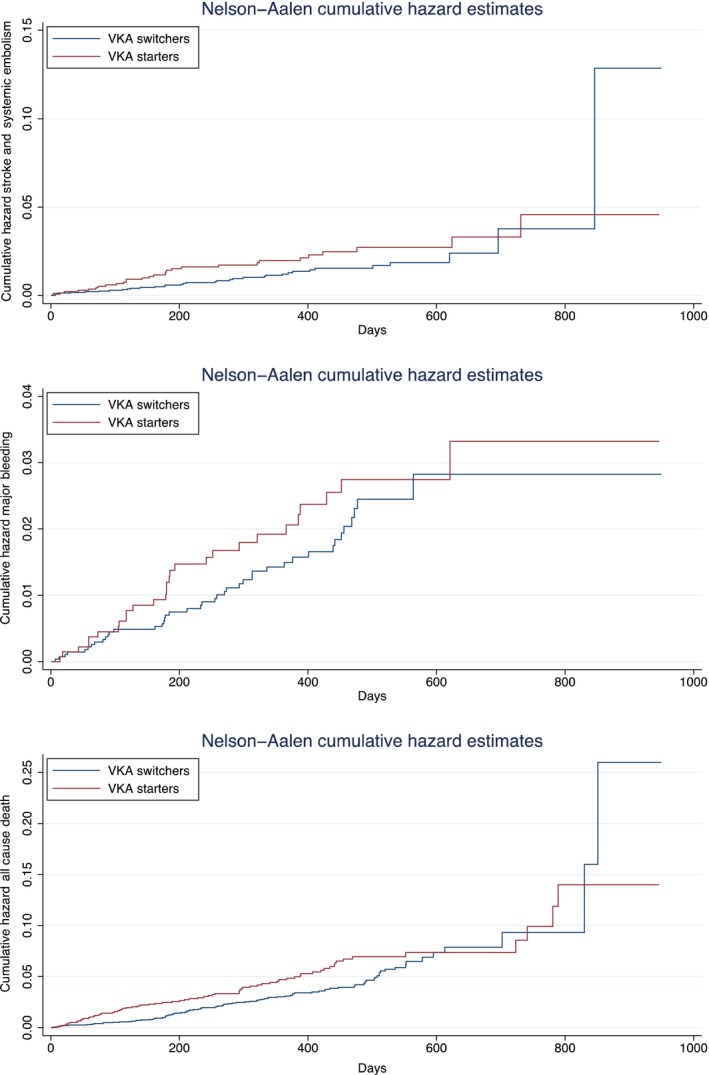
Nelson‐Aalen cumulative hazard ratios of stroke/SE, major bleeding, and all‐cause death. SE, systemic thromboembolism; VKA, vitamin K antagonists

On multivariable Cox regression analysis, with “VKA switchers” used as reference, “VKA new starters” had nonsignificant risks of stroke/SE, major bleeding, and all‐cause death in Model I and Model II (Figure 1). Kaplan‐Meier survival curves did not show significant difference in stroke/SE **(** *P* = .09**)**, major bleeding **(** *P* = .28**),** and all‐cause mortality **(** *P* = .06**)** between the two groups (Figure 2).

## 4 DISCUSSION

In this ancillary analysis of the AMADEUS and BOREALIS trials, VKA “new starters” had a nonstatistically significant risk of stroke/SE and similar rates of major bleeding and all‐cause death compared with VKA “switchers”, after adjusting for associated comorbidities and risk factors. This is contrary to the perception that “new starters” and “switchers” were patients at higher risk of adverse outcomes. The strength of this analysis is the use of pooled individual patient data from two randomized, open‐label trials, with negligible subjective intention of switching between OACs and trial‐adjudicated outcomes.

With the development of NOACs and thus increased switching from VKA to NOACs, benefits and risks among OAC switching need to be carefully assessed.^14^ Relatively lower effectiveness and safety of “switchers” from VKA to NOACs compared to NOAC “new starters” have been reported, and have questioned whether the switching to NOACs is a marker of poor adherence and higher comorbidity.^8^, ^10^, ^15^, ^16^ As patients were randomly allocated to the study groups in the randomized trials, these concerns on adherence and comorbidities are reduced in the current analysis.

One previous study,^16^ mimicking a randomized study using a marginal structural model analysis, pointed out that no other bleeding risk except for gastrointestinal bleeding risk increased in patients who switched from VKA to dabigatran. The above results were broadly consistent with the large randomized trial with dabigatran, the Randomized Evaluation of Long‐Term Anticoagulant Therapy (RE‐LY) clinical trial.^3^ Subsequently, results of the RE‐LY trial were the basis of dabigatran approval by FDA.^17^

Previous “real‐world” studies suggested that the benefits appeared to be decreased in NOAC switchers, with the assumption of poor compliance or residual confounding from comorbidities among NOACs switchers.^9^, ^10^ In the present ancillary pooled analysis of AMADEUS and BOREALIS trials on a group of VKA‐treated patients only, those who were VKA starters had similar risks of stroke/SE, major bleeding, and all‐cause death compared with VKA switchers. The main difference of the present study compared with previous comparisons lies in the fact that there was no drug switching, but not whether they had prior VKA treatment history before their strict trial‐related follow‐up. This meant that benefits were not reduced and risks were not increased by prior VKA treatment history.

One point worth mentioning is that the higher risk of all‐cause mortality rates in “VKA new‐starters” was caused by higher burden of cardiovascular diseases. One possible reason could be that they were more likely to take antiplatelet agents (aspirin, clopidogrel) as their first antithrombotic choice, but not warfarin before randomization. However, this significant difference disappeared after multivariate adjustment.

### 4.1 Switchers and starters from randomized trials

Studies are considered as having loss of precision if they only focus on new users in the evaluation of comparative effectiveness.^18^ In contrast, bias is increased by confounding in studies mainly focused on switchers. Randomized trials were composed of both starters and switchers in the two different trials at randomization, but this balance would be disrupted if comparisons were performed between switchers and starters within drugs. Indeed, previous unadjusted meta‐analysis of VKA arms of randomized trials showed an increased risk of thromboembolism in VKA starters compared to VKA switchers.^19^ Two Cox regression models were therefore used to improve the accuracy of comparisons; subsequently, our analysis showed that switchers did not show any inferiority of effectiveness or safety in the two comparison settings (VKA switchers vs VKA new starters). Although new starters had an increased burden of cardiovascular diseases, leading to higher cardiovascular and all‐cause death rates, the safety and efficacy were not significantly different after multivariate adjustment.

### 4.2 Limitations

There are several limitations for this study. First, this study lacked information of control quality of previous warfarin use and international normalised ratio (INR) values in bridging, 14 though labile INR was not the reason for the drug switching and switching between VKAs in both trials was without subjective intention. Also, we recognize some differences in clinical characteristics between the two patient groups; nonetheless, these were adjusted in our Cox Model II. Second, information on anticoagulation bridging treatment (if any) was unavailable for both studies, which made us unable to provide information whether the current results were associated with overlapped treatment or switching interval. Also, we did not have information on the OAC previously used in VKA switchers. Nevertheless, the negative results from the comparisons between VKA switchers and new starters help clarify their similar safety profile.

## 5 CONCLUSIONS

In this post hoc analysis of clinical trial patients with AF, “new starters” and “switchers” for VKA initiation had nonstatistically significant rates of trial‐adjudicated outcomes of thromboembolism, major bleeding, and all‐cause mortality. This is contrary to the perception of healthcare professionals that “new starters” and “switchers” were patients at higher risk of adverse outcomes.

## CONFLICT OF INTEREST

GYHL: Consultant for Bayer/Janssen, BMS/Pfizer, Biotronik, Medtronic, Boehringer Ingelheim, Novartis, Verseon and Daiichi‐Sankyo. Speaker for Bayer, BMS/Pfizer, Medtronic, Boehringer Ingelheim, and Daiichi‐Sankyo. No fees are directly received personally. The other authors declare no Conflict of Interests for this article”.
