# Supplementary material for: Differences in outcomes between oral anticoagulation “new starters” and “switchers” in patients with nonvalvular atrial fibrillation: A pooled analysis of the AMADEUS and BOREALIS trials
Source: J Arrhythm. 2019 Nov 11;35(6):815–20. doi: 10.1002/joa3.12255 (PMC6898535; doi:10.1002/joa3.12255)
Supplement: Supplementary file 1 [file JOA3-35-815-s001.doc]

**ONLINE SUPPLEMENT**

**Differences in outcomes between oral anticoagulation ‘new starters’ and ‘switchers’ in patients with non-valvular atrial fibrillation: A pooled analysis of the AMADEUS and BOREALIS trials**

**Short title:** Outcomes in ‘new starters’ and ‘switchers’

Ying Bai, PhD ; Alena Shantsila, PhD; Gregory Y.H.Lip, MD, FRCP

| Supplemental Methods | Page 2 |
| --- | --- |
| Supplemental Tables | Page 3 |
|  |  |

**Supplemental Methods**

The AMADEUS trial^1^ was a multi-center, randomized, open-label non-inferiority study with blinded assessment of outcome that compared fixed-dose idraparinux with conventional anticoagulation by dose-adjusted oral VKA therapy for the prevention of thromboembolism in AF patients. Eligible patients had ECG-documented non-valvular AF and an indication for long-term anticoagulation, based on the presence of at least 1 of the following risk factors: previous ischemic stroke, transient ischemic attack or systemic embolism, hypertension requiring drug treatment, left ventricular dysfunction, age >75 years, or age 65 to 75 years with either diabetes mellitus or symptomatic coronary artery disease (CAD). Patients with severe renal failure (calculated creatinine clearance of <10 mL/min) were excluded.

The BOREALIS trial^2^ was a multicenter, randomized, double-blind, double-dummy, non-inferiority trial comparing idrabiotaparinux (or its placebo) and dose-adjusted warfarin (or its placebo) for the prevention of thromboembolism in patients with AF. Eligibility criteria were permanent, persistent or paroxysmal, non-valvular, electrocardiogram (ECG) documented AF with an indication for long-term VKA therapy based on the presence of previous ischemic stroke, transient ischemic attack, or systematic thromboembolism and/or at least two of the following risk factors: hypertension requiring drug treatment; moderately or severely impaired left ventricular function and/or heart failure; age ≥ 75 years; or diabetes mellitus. Patients with severe renal failure (creatinine clearance < 30 mL/min), uncontrolled hypertension (systolic blood pressure > 180 mm Hg and/or diastolic blood pressure > 110 mm Hg) were not included.

| **Supplemental Table I.** Baseline Characteristics of VKA users in AMADEUS and BOREALIS datasets | | | | | | |
| --- | --- | --- | --- | --- | --- | --- |
|  | AMADEUS | |  | BOREALIS | |  |
|  | starter | switcher | P value | starter | switcher | P value |
| Total patients | 552 | 1741 |  | 831 | 1045 |  |
| Age (years) | 71.33±9.75 | 69.83±8.80 | <0.001 | 68.42±9.73 | 69.52±9.76 | 0.02 |
| Categorized by group |  |  |  |  |  |  |
| Age≥75 | 239(43.3) | 585(33.6) | <0.001 | 234(28.2) | 337(32.3) | 0.09 |
| Age 65-74 | 184(33.3) | 698(40.1) |  | 313(37.7) | 392(37.6) |  |
| Age <65 | 129(23.4) | 458(26.3) |  | 284(34.2) | 315(30.2) |  |
| Gender female | 230(41.7) | 561(32.2) | <0.001 | 362(43.6) | 377(36.1) | 0.001 |
| AF type |  |  | <0.001 |  |  | 0.46 |
| Paroxysmal AF | 251(46.1) | 562(32.3) |  | 228(27.6) | 268(25.7) |  |
| Persistent AF | 53(9.7) | 161(9.3) |  | 139(16.9) | 195(18.7) |  |
| Permanent AF | 241(44.2) | 1017(58.5) |  | 458(55.2) | 581(55.7) |  |
| BMI (kg/m^2^) | 28.25±5.34 | 28.30±5.74 | 0.007 | 29.41±5.74 | 30.48±6.24 | <0.001 |
| Hypertension | 437(79.2) | 1327(76.2) | 0.15 | 796(95.8) | 968(92.6) | 0.004 |
| Prior TIA/Stroke/SE | 166(30.1) | 409(23.5) | 0.002 | 220(26.5) | 331(31.7) | 0.01 |
| Diabetes Mellitus | 93(16.9) | 357(20.5) | 0.06 | 242(29.1) | 388(37.1) | <0.001 |
| LV dysfunction | 111(20.1) | 432(24.8) | 0.02 | 569(68.5) | 527(50.4) | <0.001 |
| Coronary artery disease | 167(30.3) | 551(31.7) | 0.54 | 385(51.0) | 382(38.4) | <0.001 |
| Concomitant treatment |  |  |  |  |  |  |
| Aspirin | 185(33.5) | 194(11.1) | <0.001 | 413(49.7) | 196(18.8) | <0.001 |
| Clopidogrel or Ticagrelor | 24(4.4) | 14(0.8) | <0.001 | 21(2.5) | 16(1.5) | 0.12 |
| Other antiplatelet | 4 (0.7) | 8(0.5) | 0.45 | 6(0.7) | 2(0.2) | 0.08 |
| TTR (%) | 49.56±19.01 | 59.68±19.76 | <0.001 | 53.68±24.78 | 60.86±24.51 | <0.001 |
| Baseline creatinine clearance(ml/min) | 98.08±27.40 | 96.97±25.19 | 0.38 | 79.35±32.33 | 81.96±34.52 | 0.10 |
| Categorized by group |  |  |  |  |  |  |
| <30 | 0(0) | 2(0.12) | 0.61? | 0(0) | 3(0.29) | 0.25 |
| 30-50 | 2(0.37) | 3(0.17) |  | 144(17.45) | 160(15.47) |  |
| 50-80 | 135(24.91) | 405(23.45) |  | 336(40.73) | 414(40.04) |  |
| ≥80 | 405(74.72) | 1317(76.26) |  | 345(41.82) | 457(44.20) |  |
| CHA_2_DS_2_-VASc score | 3.68±1.61 | 3.40±1.52 | <0.001 | 4.32±1.38 | 4.20±1.38 | 0.055 |
| 0 | 0(0) | 0(0) | <0.001 | 0(0) | 0(0) | 0.42 |
| 1 | 43(7.8) | 157(9.0) |  | 0(0) | 1(0.1) |  |
| 2 | 105(19.0) | 377(21.7) |  | 62(7.5) | 99(9.6) |  |
| 3 | 109(19.8) | 455(26.1) |  | 188(22.8) | 236(22.8) |  |
| 4 | 132(23.9) | 353(20.3) |  | 226(27.5) | 307(29.7) |  |
| 5 | 84(15.2) | 239(13.7) |  | 186(22.6) | 219(21.2) |  |
| ≥6 | 79(14.3) | 160(9.2) |  | 161(19.6) | 171(16.6) |  |
| HAS-BLED score | 2.26±1.00 | 1.70±0.95 | <0.001 | 2.06±1.06 | 1.82±1.01 | <0.001 |
| 0 | 22(4.0) | 157(9.0) | <0.001 | 39(4.7) | 84(8.1) | <0.001 |
| 1 | 93(16.9) | 596(34.2) |  | 218(26.5) | 326(31.6) |  |
| 2 | 215(39.0) | 656(37.7) |  | 314(38.2) | 373(36.2) |  |
| 3 | 169(30.7) | 282(16.2) |  | 168(20.5) | 192(18.6) |  |
| 4 | 46(8.3) | 43(2.5) |  | 73(8.9) | 55(5.3) |  |
| ≥5 | 6(1.1) | 7(0.4) |  | 10(1.2) | 2(0.2) |  |

Data were presented as mean ± SD or percentage of actual patients number; AF, atrial fibrillation; BMI, body mass index; CHA2DS2-VASc, congestive heart failure,1 point; hypertension, 1 point; age ≥ 75 years,2 points; diabetes mellitus,1 point; stroke, 2 pints; vascular disease, 1 point; age from 65 to 74 years, 1 point; and female sex, 1 point; HAS-BLED, uncontrolled [hypertension](https://en.wikipedia.org/wiki/Hypertension)(>160 mmHg systolic), 1 point; [abnormal renal function](https://en.wikipedia.org/wiki/Renal_Disease), 1 point; or [abnormal liver function](https://en.wikipedia.org/wiki/Liver_Disease), 1 point; Prior history of stroke, 1 point; Prior major bleeding or predisposition to bleeding, 1 point; labile INR, 1 point; age > 65 years, 1 point; prior Alcohol or Drug Usage History (≥ 8 drinks/week), 1 point; Medication Usage Predisposing to Bleeding: (Antiplatelet agents, NSAIDs), 1 point; LV, left ventricle; SE, systemic thromboembolism; TIA, transient ischemic attack; TTR, time in therapeutic range; VKA, vitamin-K antagonists.

| **Supplemental Table II.** Event rates for efficacy and safety outcomes in VKA users | | | | | | | |
| --- | --- | --- | --- | --- | --- | --- | --- |
|  |  | | | | | |  |
|  | New starter | | Switcher | | |  | |
|  | N | Event rate  (per 100 p-y) | N | Event rate  (per 100 p-y) | P value | |  |
| **Efficacy** |  |  |  |  |  | |  |
| Stroke/SE | 29 | 2.04(1.37-2.93) | 35 | 1.29(0.90-1.79) | 0.07 | |  |
| Ischemic stroke | 17 | 1.19(0.70-1.91) | 26 | 0.96(0.63-1.40) | 0.48 | |  |
| Non-ischemic stroke | 8 | 0.56(0.24-1.10) | 7 | 0.26(0.10-0.53) | 0.12 | |  |
| Non-CNS systemic embolism | 4 | 0.28(0.08-0.72) | 3 | 0.11(0.02-0.32) | 0.21 | |  |
| VTE | 3 | 0.21(0.04-0.61) | 7 | 0.26(0.10-0.53) | 0.77 | |  |
| Myocardial infarction | 7 | 0.49(0.20-1.01) | 11 | 0.40(0.20-0.72) | 0.69 | |  |
| **Safety** |  |  |  |  |  | |  |
| Any clinically relevant bleeding | 144 | 10.7(9.02-12.6) | 272 | 10.55(9.34-11.89) | 0.9 | |  |
| Major bleeding | 28 | 1.97(1.31-2.84) | 42 | 1.55(1.12-2.09) | 0.02 | |  |
| ICH | 10 | 0.70(0.34-1.29) | 14 | 0.51(0.28-0.86) | 0.46 | |  |
| **Deaths** |  |  |  |  |  | |  |
| All-cause death | 70 | 4.88(3.8-6.16) | 96 | 3.51(2.84-4.29) | 0.036 | |  |
| Fatal stroke | 7 | 0.49(0.20-1.00) | 5 | 0.18(0.06-0.43) | 0.08 | |  |
| Cardiovascular death | 36 | 2.51(1.76-3.47) | 44 | 1.61(1.17-2.16) | 0.047 | |  |

CNS, central nervous system; ICH, Intracranial haemorrhage; N, number; SE, systemic thromboembolism; VKA, vitamin-K antagonists; VTE, Venous thromboembolism.

**References**

1. Amadeus I, Bousser MG, Bouthier J, et al. Comparison of idraparinux with vitamin K antagonists for prevention of thromboembolism in patients with atrial fibrillation: a randomised, open-label, non-inferiority trial. *Lancet.* 2008;371(9609):315-321.

2. Buller HR, Halperin J, Hankey GJ, Pillion G, Prins MH, Raskob GE. Comparison of idrabiotaparinux with vitamin K antagonists for prevention of thromboembolism in patients with atrial fibrillation: the Borealis-Atrial Fibrillation Study. *J Thromb Haemost.* 2014;12(6):824-830.
